# Investigating papillary thyroid cancer risk factors among women living at the central region of Iran: a case–control study

**DOI:** 10.1186/s12902-025-01833-3

**Published:** 2025-01-16

**Authors:** Hamed Ghoshouni, Saeed Hosseini, Akram Ghadiri-Anari, Reyhaneh Azizi, Masoud Rahmanian, Narjes Hazar

**Affiliations:** 1 Cardiovascular Epidemiology Research Center, Rajaie Cardiovascular Institute, Tehran, Iran; 2 Department of Epidemiology, School of Public Health, Iran University of Medical Sciences, Tehran, Iran; 3 Center for Healthcare Data Modeling, Department of Biostatistics and Epidemiology, School of Public Health, Shahid Sadoughi University of Medical Sciences, Yazd, Iran; 4 Diabetes Research Center, Shahid Sadoughi University of Medical Sciences, Yazd, Iran

**Keywords:** Thyroid Cancer, Papillary, Risk factors, Contraceptives, Oral, Case–control studies, Iran

## Abstract

**Background:**

The etiology of thyroid cancer especially in women in not well recognized in Yazd, at the center of Iran. The aim of present study was to investigate the risk factors of thyroid cancer among women living in this province.

**Methods:**

The present study was carried out as a case–control study, comprising women diagnosed with papillary thyroid cancer (PTC) as the case group, along with two distinct control groups sourced from different origins (i.e., relatives and non-relatives) between 2020 and 2022. Data pertaining to several risk factors including demographic characteristics, reproductive variables, medical history related to thyroid and non-thyroid ailments, exposure to head and neck radiation, as well as familial cancer history, was collected from all participants. Binary logistic regression was utilized to discover risk and protective factors.

**Results:**

In present study, 77 individuals participated in the case group, 76 in the relative control group and 72 in the non-relative control group. The history of OCP use and exposure to head and neck radiation were remained in the model as risk factors in all three case‒relative control (OR = 6.65, 95%CI: 2.53‒17.49; *P*-value < 0.001), case‒non-relative control (OR = 6.32, 95%CI: 2.14‒18.70; *P*-value = 0.001) and case‒total control comparisons (OR = 6.66, 95%CI: 2.84‒15.64; *P*-value < 0.001).

**Conclusion:**

The OCP use as well as exposure to head and neck radiation were determined to be strong or relatively strong risk factors in both case‒relative control and case‒non-relative control comparisons. Consequently, it seems these two factors represent genuine risk factors for papillary thyroid cancer.

**Supplementary Information:**

The online version contains supplementary material available at 10.1186/s12902-025-01833-3.

## Introduction

Thyroid cancer is a relatively rare neoplasm worldwide, accounting for approximately 1–5% of all cancers in women and less than 2% of all cancers in men [1]. Although thyroid cancer is relatively rare, it is the most common endocrine malignancy worldwide [2] and it is estimated that thyroid cancer will be the fourth most common diagnosis of all cancers by 2030 [3]. This cancer is the second most common cancer in women and the sixth most common cancer in both sexes in the Iranian population [4].

Thyroid cancer occurs two to four times more often in women than in men [5] and is rare in children and adolescents, even though the risk of malignancy of thyroid nodules is higher in young people [6]. Thyroid carcinoma may originate from thyroid follicular cells or parafollicular cells (C cells). Follicular cell cancer is divided into different types, the most common type of which is papillary thyroid carcinoma (PTC) (75–85% of cases) [7].

Some studies relate the increase in the incidence of thyroid cancer to the use of more accurate and sensitive diagnostic methods [8, 9]. However, some others suggest that better diagnostic technology cannot fully account for this increment [10] and other factors such as environmental factors, lifestyle, and related diseases and disabilities can play a substantial role [7, 11]. Modifiable risk factors, including smoking, alcohol consumption, and obesity, are among the risk factors of many cancers [12–14]. However, their relationship with thyroid cancer in different studies exert different results. Some cohort studies have shown an inverse association between the risk of thyroid cancer with tobacco use or moderate alcohol consumption [15], while some have found no statistical association [16]. Some studies have demonstrated a protective effect of alcohol in thyroid cancer [17, 18], while others have not revealed this type of relationship [19].

Regarding the role of body mass index, it seems that a higher body mass index (BMI) is associated with a more advanced and histopathologically aggressive stage of thyroid cancer at the time of diagnosis [20, 21], but not all studies agree that it increases the risk of developing thyroid cancer [22, 23].

According to the results of a robust meta-analysis, a history of benign thyroid diseases especially goiter and both hypo and hyperthyroidism has been connected with an increased risk of thyroid cancer [24].

Several evidences show that female sex hormones play a role in developing thyroid cancer. In detail, research on mouse models of thyroid cancer has highlighted the role of estrogen as a promoter in the development of thyroid tumors [25]. Meanwhile, research results demonstrated that high serum estrogen may be considered as a risk factor for PTC in human [26].

In addition to the mentioned cases, exposure to X-ray radiation is one of the most important risk factors, so that exposure to X-ray radiation, especially in the neck region, is the most accepted risk factor for thyroid cancers in the literature [27–30].

The results of Iranian population-based cancer registry between 2014–2018 at the national and also local (Yazd province) level showed that thyroid cancer had a significant incidence in women, while it was not the case in men. The results of these reports demonstrated that thyroid cancer was the first common cancer among women in Yazd province in 2016, the second most common cancer in 2015 and 2017–18, and the third most common cancer in 2014. In addition, in 2014–2018, Yazd had the highest incidence of female thyroid cancer among other provinces of Iran [4, 31–34]. However, thyroid cancer in men had never been among the first 5 cancers, and it had been ranked 7th and below in the mentioned reports. Accordingly, it seems necessary to find the risk factors of this cancer among women living in Yazd in order to design and conduct effective preventive interventions. The purpose of this research was to identify some risk factors of thyroid cancer in the population of women living in Yazd province.

## Materials and methods

The present study was conducted as a case–control study with two different control groups from different sources (relative and non-relative control groups) in Yazd province, the central region of Iran in 2020–2022 (the location of Yazd is provided in the supplementary file). The case group was selected from female patients with PTC, the relative control group consisted of the patient’s sister/mother, and the non-relative control group was selected from mothers /sisters of students of Shahid Sadoughi University of Medical Sciences, Yazd.

### Inclusion criteria

Inclusion criteria for the case group included: female, residence for at least 10 years in one of the cities of Yazd province, and a definite case of thyroid cancer with papillary histology that had been diagnosed within one month prior to participation in the study (incident case).

The inclusion criteria for the relative control group included a non-twin sister (the mother in the absence of any sister) and not suffering from any type of cancer, and the inclusion criteria for the non-relative control group included being a woman, living for at least 10 years in one of the cities of Yazd province, and not suffering from any type of malignancy.

### Sample size

To determine the sample size, below formula, which is one of the appropriate formulas in case–control studies, was used.

$$\mathrm n=\frac{\mathrm k+1}{\mathrm k}\times\frac{\left({\mathrm z}_{1-{\displaystyle\frac{\mathrm\alpha}2}}+{\mathrm z}_{1-\mathrm\beta}\right)}{\left(\mathrm{lnOR}\right)^2\overline{\mathrm P}\left(1-\overline{\mathrm P}\right)}$$

In this formula, $\overline{\text{P} }$ is the average exposure to the risk factor in the case and control group, which was calculated as $\overline{\text{P} }=\frac{{\text{P}}_{1}+\left({\text{P}}_{2}\right.\times \left.\text{K}\right)}{1+\text{K}}$. K was also the ratio of the size of the control group to the case group. In this study, α = 5%, β = 0.1, and since we planned the size of the control group to be twice the size of the case group, so K = 2. According to the results of Karkoobi et al. study [35], the prevalence of family history of thyroid cancer was 30.6 in the case group and 11.7 in the control group, and the OR was 1.98. Considering the above-mentioned items, the required sample size in each group was 64, and after taking 15% attrition into account, the final sample size was determined to be 75 for each group.

### Sampling method

After obtaining the ethics code for the current study, each of the three collaborating physicians, as soon as they diagnosed a female patient with thyroid cancer of the PTC type based on the pathology report, they talked to the patient about the current study, and after obtaining their verbal consent by relevant physician, the patient entered the research as the case group member. The relative control group selected from the non-twin sister of the patient (or the mother if the patient did not have an available sister), and the non-relative control group was selected from mothers or sisters of students of Shahid Sadoughi University of Medical Sciences, Yazd through convenience sampling.

### Data collection tool and data gathering method

The data required for the present study were collected by a data collection form designed by the research team. The questions in this form were designed based on the risk factors extracted based on literature review including age, education, occupation, marital status, weight, height, blood group, age at first menstruation period, menstruation regularity, menopause, history of OCP use, pregnancy, abortion, live birth, benign thyroid mass, Goiter, hyperthyroidism, hypothyroidism, history of other diseases, head and neck radiation and family history of cancer. The English language version of the data collection form has been provided as a supplementary file.

Since data gathering phase of current study had fallen within the COVID-19 pandemics, it was not possible to invite the patients in the case group as well as the control group participants to complete the questionnaire in order to maintain their health. Therefore, the data gathering form was designed electronically. It was prepared in such a way that the answer to the first question was necessary to go to the second question, and this mode existed for all questions.

After selecting the patient and obtaining her consent by the relevant physician, her contact number was given to a female associate of the project. She talked with the patient during a phone call and talked about the present project as well as the content of and how to complete the electronic questionnaire. She also requested them to talk to their sister/mother and after obtaining their consent, provide her with their contact number. Then, necessary explanations were given to the relative control group during a phone call. Also, for the non-relative control group, the sister/mother of the students of Yazd Shahid Sadougi University of Medical Sciences, who agreed to participate in this study were given full explanation about the study. After making the necessary arrangements, the link of the questionnaire was sent to each participant through SMS and they were reminded every week until the completion of the questionnaire. 2.5

### Data analysis method

The collected data was analyzed using SPSS software version 20. Mean and standard deviation were used for descriptive statistics of quantitative variables and frequency was used for description of qualitative variables. In order to compare the quantitative variables between the case group and every control group, the Independent Sample T Test was used. Also, chi-square test was used to compare the qualitative variables between groups. Meanwhile, Binary logistic regression was employed to explore the association between group membership (case/control) as dependent variable and other factors as independent variables. In order to get the best result, each independent variable with *P*-value less than 0.2 in the univariate analysis was included in the Backward multivariable logistic regression model. The mentioned model with adjusted odds ratio (with 95% confidence level) was utilized to find predictive factors of PTC. This model constitutes an iterative methodology that commences with a comprehensive model encompassing all desired variables. At each successive stage of the analysis, the variables exhibiting the least significance are systematically removed from the model. This procedure persists until a final model with a part of input variables is achieved that offers the most comprehensive elucidation of the data.

Since there were three different control groups, three separate Backward multivariable logistic regression models were created. The first model was for relative control group in which age, BMI, education, job, marriage, irregular menstruation, OCP consumption, pregnancy history, abortion, live birth history, thyroid nodule, hypothyroidism, other disease history and head & neck radiation were included and age, irregular menstruation, OCP consumption, pregnancy history, thyroid nodule and head & neck radiation were remained after completion the analysis. The second model for the non-relative control group was executed with BMI, education, job, blood group, irregular menstruation, menopause, OCP consumption, abortion, thyroid nodule, goiter history, hypothyroidism, head & neck radiation and family cancer history and ultimately narrowed down to job, blood group, OCP consumption, head & neck radiation and family cancer history. The third model which used the data of total control group, started with age, BMI, education, job, blood group, irregular menstruation, OCP consumption, abortion, thyroid nodule, hypothyroidism and head & neck radiation and finally ended with age, education, irregular menstruation, OCP consumption, thyroid nodule and head & neck radiation. A *P*-value less than 0.05 was regarded as statistically significant.

## Results

In present study, 77 individuals participated in the case group, 76 in the relative control group and 72 in the non-relative control group. The average age in the case group at the time of diagnosis was 37.34 ± 7.6 and the average age of the relative, non-relative and total control groups was 42.75 ± 11.4, 37.93 ± 6.4, and 40.41 ± 9.6 respectively. Of the total participants, 39 individuals (50.6%) in the case group and 50 (65.8%), 10 (13.9%) and 60 (40.5%) individuals in the relative, non-relative and total control groups were housekeeper. In terms of education, about half of the people in the case and relative control groups had college education, while this rate was about 90% in the non-relative control group (*P*-value: 0.001). Descriptive statistics of all participants has been presented in Table 1.

**Table 1:** Descriptive characteristics of participants in case and control groups & analytical results between case and each control group

**Variable**	**Category**	**Case**	**Control (total)**	***P*-value** Case & total control	**Control (relative)**	***P*-value** Case & relative control	**Control (non-relative)**	***P*-value** Case & non-relative control
Age, Year		37.34 ± 7.6	40.41 ± 9.6	0.010	42.75 ± 11.4	0.001	37.93 ± 6.4	0.611
Age of menarche, Year		13.48 ± 1.6	13.67 ± 1.3	0.341	13.62 ± 1.3	0.558	13.72 ± 1.3	0.310
BMI, Kg/M^2^		26.85 ± 4.5	25.77 ± 3.4	0.068	25.85 ± 3.8	0.141	25.69 ± 3.0	0.065
Education, N(%)	Under Diploma	13 (16.9)	26 (17.6)	0.127	23 (30.3)	0.116	3 (4.2)	0.001
	Diploma	18 (23.4)	17 (11.5)		11 (14.5)		6 (8.3)	
	BSc/BA	30 (39.0)	65 (43.9)		32 (42.1)		33 (45.8)	
	MSc/MA & above	16 (20.8)	40 (27.0)		10 (13.2)		30 (41.7)	
Job	House keeper	39 (50.6)	60 (40.5)	0.012	50 (65.8)	0.110	10 (13.9)	< 0.001
	Self employed	16 (20.8)	17 (11.5)		8 (10.5)		9 (12.5)	
	Employee	22 (28.6)	71 (48.0)		18 (23.7)		53 (73.6)	
Marriage	Married	70 (90.9)	139 (93.9)	0.405	74 (97.4)	0.090	65 (90.3)	0.895
Blood group	A	21 (27.3)	37 (25)	0.073	20 (26.3)	0.541	17 (23.6)	0.012
	B	13 (16.9)	46 (31.1)		20 (26.3)		26 (36.1)	
	AB	10 (13)	22 (14.9)		9 (11.8)		13 (18.1)	
	O	33 (42.9)	43 (29.1)		27 (35.5)		16 (22.2)	
Irregular menstruation	Yes	28 (36.4)	30 (20.3)	0.009	12 (15.8)	0.004	18 (25)	0.133
Menopause	Yes	12 (15.6)	18 (12.2)	0.474	14 (18.4)	0.640	4 (5.6)	0.048
OCP consumption	Yes	21 (27.3)	11 (7.4)	< 0.001	7 (9.2)	0.004	4 (5.6)	< 0.001
Pregnancy history	Yes	63 (81.8)	128 (86.5)	0.354	72 (94.7)	0.013	56 (77.8)	0.539
Abortion	Yes	17 (22.1)	19 (12.8)	0.073	10 (13.2)	0.148	9 (12.5)	0.124
Live birth history	Yes	60 (77.9)	118 (79.7)	0.752	68 (89.5)	0.053	50 (69.4)	0.239
Thyroid nodule	Yes	33 (42.9)	27 (18.2)	< 0.001	15 (19.7)	0.002	12 (16.7)	0.001
Goiter history	Yes	9 (11.7)	18 (12.2)	0.917	14 (18.4)	0.244	4 (5.6)	0.185
Hyperthyroidism	Yes	8 (10.4)	17 (11.5)	0.804	5 (6.6)	0.398	12 (16.7)	0.261
Hypothyroidism	Yes	30 (39.7)	42 (28.4)	0.106	22 (28.9)	0.191	20 (27.8)	0.149
Other disease history	Yes	14 (18.2)	33 (22.3)	0.471	21 (27.6)	0.164	12 (16.7)	0.808
Head & neck radiation	Yes	34 (44.2)	28 (18.9)	< 0.001	12 (15.8)	< 0.001	16 (22.2)	0.005
Family cancer history	Yes	37 (48.1)	‒	‒	‒	‒	10 (13.9)	< 0.001

Examining the distribution of demographic variables and some predictive factors through univariate analysis between two case and relative control groups revealed that age, irregular menstruation, history of OCP use, pregnancy, benign thyroid mass and head and neck radiation have a significant relationship with PTC (Table 1). In the multivariable logistic analysis using the Backward method, the history of OCP use (OR: 8.31; 95% CI: 2.52‒27.39), no history of pregnancy (OR: 4.52; 95% CI: 1.05‒19.34), history of benign thyroid mass (OR: 8.57; 95%CI: 2.94‒24.99) and history of head and neck radiography (OR: 6.65; 95%CI: 2.53‒17.49) were recognized as risk factors and increasing age (OR: 0.91; 95%CI: 0.89‒ 0.99) as a protective factor for the PTC occurrence (Table 2). The model fitness based on Nagelkerke R^2^ was calculated as 0.518.

**Table 2:** Crude and adjusted OR with 95% confidence interval for the association of predictive factors with PTC, relatives control group

Variable	Category	Crude OR	*P*-value	Adjusted OR	*P*-value
Age, Year	‒	0.94 (0.91‒0.97)	0.001	0.91 (0.89‒0.99)	0.021
Irregular menstruation	Yes	3.04 (1.40‒6.59)	0.005	0.35 (0.12‒1.02)	0.056
	No	1	‒	1	‒
OCP consumption	Yes	3.69 (1.46‒9.32)	0.006	8.31 (2.52‒27.39)	0.001
	No	1	‒	1	‒
Thyroid nodule	Yes	3.05 (1.48‒6.28)	0.003	8.57 (2.94‒24.99)	< 0.001
	No	1	‒	1	‒
Head & neck radiation	Yes	4.21 (1.96‒9.04)	< 0.001	6.65 (2.53‒17.49)	< 0.001
	No	1	‒	1	‒
Pregnancy history	No	4.00 (1.25‒12.77)	0.019	4.52 (1.05‒19.34)	0.042
	Yes	1	‒	1	‒

In the study of the relationship between the case group and the non-relative control group, the variables of education, occupation, blood group, menopause, history of OCP use, benign thyroid mass, head and neck radiography and family cancer history showed a significant relationship with PTC (Table 1). Based on the results of multivariable logistic analysis using the backward method, housekeeping (OR: 40.88; 95%CI: 9.19‒181.85), history of OCP use (OR: 43.71; 95%CI: 3.08‒619.36), family cancer history (OR: 11.98; 95%CI: 3.56‒40.30) and history of head and neck radiography (OR: 6.32; 95%CI: 2.14‒18.70) remained in the model as a risk factor and blood group A (OR: 0.19; 95%CI: 0.05‒0.79)) as a protective factor for PTC (Table 3). The model fitness based on Nagelkerke R^2^ was calculated as 0.648.

**Table 3:** Crude and adjusted OR with 95% confidence interval for the association of some prognostic factors with PTC, non-relatives control group

Variable	Category	Crude OR	*P*-value	Adjusted OR	*P*-value
Job	House keeper	9.39 (3.99‒22.07)	< 0.001	40.88 (9.19‒181.85)	< 0.001
	Freelance	4.28 (1.64‒11.14)	0.003	2.52 (0.54‒11.63)	0.234
	Employee	1	‒	1	‒
Blood group	A	0.59 (0.25‒1.43)	0.251	0.19 (0.05‒0.79)	0.022
	B	0.24 (0.09‒0.59)	0.002	6.58 (0.70‒61.22)	0.097
	AB	0.37 (0.13‒1.03)	0.058	2.97 (0.76‒11.53)	0.115
	O	1	‒	1	‒
OCP consumption	Yes	6.37 (2.06‒19.66)	0.001	43.71 (3.08‒619.36)	0.005
	No	1	‒	1	‒
Head & neck radiation	Yes	2.76 (1.35‒5.65)	0.005	6.32 (2.14‒18.70)	0.001
	No	1	‒	1	‒
Family history of cancer	Yes	5.73 (2.56‒12.81)	< 0.001	11.98 (3.56‒40.30)	< 0.001
	No	1	‒	1	‒

In examining the relationship between the case group and the total control group, all the variables of age, occupation, regular menstruation, history of OCP use, history of benign thyroid mass, and history of head and neck radiography showed a significant relationship with the occurrence of PTC (Table 1). The results of multivariable logistic regression revealed that education of below diploma (OR:5.62; 95%CI:1.43‒22.06) and diploma (OR:4.22; 95%CI:1.26‒14.19), irregular menstruation (OR:2.90; 95%CI:1.21) ‒6.95), history of OCP use (OR:7.09; 95%CI: 2.47‒20.31), history of head and neck radiography (OR:6.66; 95%CI:2.84‒15.64) and history of benign thyroid mass (OR:4.75; 95%CI:2.01‒11.25) played the role as risk factor and age (OR:0.91; 95%CI:0.96‒0.87) played the role as protective factor for PTC (Table 4). The model fitness based on Nagelkerke R^2^ was calculated as 0.480.

**Table 4:** Crude and adjusted OR with 95% confidence interval for the association of some prognostic factors with PTC, total control group

Variable	Category	Crude OR	*P*-value	Adjusted OR	*P*-value
Age, Year	‒	0.96 (0.93‒0.99)	0.018	0.91 (0.96‒0.87)	0.001
Education	Under diploma	1.25 (0.51‒3.02)	0.620	5.62 (1.43‒22.06)	0.013
	Diploma	2.64 (1.09‒6.38)	0.030	4.22 (1.26‒14.19)	0.020
	Bachelor	1.15 (0.56‒2.37)	0.698	1.59 (0.61‒4.12)	0.337
	Master & above	1	‒	1	‒
Irregular menstruation	Yes	2.24 (1.21‒4.15)	0.01	2.90 (1.21‒6.95)	0.016
	No	1	‒	1	‒
OCP consumption	Yes	4.67 (2.11‒10.32)	< 0.001	7.09 (2.47‒20.31)	< 0.001
	No	1	‒	1	‒
Thyroid nodule	Yes	3.36 (1.18‒6.21)	< 0.001	4.75 (2.01‒11.25)	< 0.001
	No	1	‒	1	‒
Head & neck radiation	Yes	3.38 (1.84‒6.23)	< 0.001	6.66 (2.84‒15.64)	< 0.001
	No	1	‒	1	‒

## Discussion

In present study, we examined the risk factors of PTC among women living in Yazd because it had the highest incidence among women's cancers in Yazd for consecutive years, while it was not the case in men. Whether or not the incidence of thyroid cancer is actually higher in women was not one of the goals of this study. However, the results of some researches demonstrated that the incidence in women is not actually higher, but more women than men undergo diagnostic tests that may detect small thyroid cancers that are unlikely to create serious problems throughout their lives [36] and this leads to overdiagnosis and overtreatment of thyroid cancer in women. In spite of this issue and considering that efforts to reduce the occurrence of these small malignancies can also prevent the psychological and financial burden imposed on female patients, we decided to investigate some risk factors raised in studies on female patients.

In present study, a specific design of case–control study called “a case control study with multiple controls from different types” was utilized in which two control groups including a relative (non-twin sister/mother) and a non-relative control groups were existed. Using this type of study helps to test different hypotheses on one hand and to check the possibility of recall bias effect on the other hand. Meanwhile, the use of relative controls can be a kind of adjustment on the genetic differences between case and control groups. Since people suffering from a serious disease as case group may remember exposure to risk factors more than individuals without that condition as control group (recall bias), using a close relative of individuals with cancer as the control group can attenuate the recall bias occurring in the study [37].

### OCP use

In this study, OCP consumption was the strongest and most important risk factor for thyroid cancer in the case‒relative control and case‒total control association, and the second most important risk factor in the case‒non-relative control association.

The results of prior research in this area exhibit discrepancies. A meta-analysis disseminated in 2015 revealed the potential of OCP consumption to diminish the susceptibility to thyroid cancer among females. The aforementioned meta-analysis stemmed from consolidating 9 cohort studies indexed in PubMed. The merit of this analysis lies in its exclusive incorporation of cohort studies while the demerit pertains to its sole reliance on the PubMed database for data retrieval [38]. An observational study conducted in Korea, compared the utilization of OCP in two groups- individuals with thyroid cancer and a control group devoid of the affliction. The impact of OCP usage on the manifestation of thyroid cancer was scrutinized through logistic regression. In this study, OCP use was a protective factor for PTC cancer, but in subgroup analysis based on age, this relationship was not remained significant, especially in women of reproductive age. The findings of this investigation identified OCP usage as a protective factor for PTC cancer; however, subsequent subgroup analysis based on age unveiled the insignificance of this association, particularly among women of reproductive age [39]. Contrarily, a study aimed at exploring the possible effects of the combination of the two steroids Ethinylestradiol (EE) and levonorgestrel (LNG) from OCPs on metastasis and angiogenic factors in BCPAP papillary thyroid cancer cell line (PTC) noted the propensity of these components to instigate metastasis from PTC [40]. Meanwhile, a separate study conducted to assess the impact of low-dose combined OCP (LD-COC) on the proliferation, apoptosis, and migration of BCPAP cell line revealed that these substances bolster cell growth and migration while diminishing cell death compared to the control cell group. Consequently, it appears that Combined Oral Contraceptives (COCs) play a role in stimulating the growth of PTC cells, indicating a robust correlation between the estrogen and progesterone constituents of COCs and the progression of thyroid cancer [41].

In present study, the OR for the association between OCP use and thyroid cancer in non-relative control group was much larger than the OR in the relative group (43.71 vs 8.31). According to the explanations given at the beginning of the discussion, the recall bias concerning this risk factor occurred in the non-relative control group. However, a fraction of the dissimilarity between two ORs may be attributed to the mediating role of genetic factors in the association between OCP consumption and thyroid cancer. Taking into account that in the relative control group whose first-degree relatives (sister/daughter) were suffering from thyroid cancer, OCP use was also a risk factor with a relatively large OR, so there is a possibility that bias or bias occurred less. and OCP use as a real risk factor for thyroid cancer. Notably, OCP utilization emerged as a risk factor with a relatively high OR in the relative control group, comprising individuals with first-degree relatives (sister/daughter) grappling with thyroid cancer, hinting at a diminished likelihood of recall bias. This underscores the plausibility of OCP usage as a real risk factor for thyroid cancer. Nevertheless, it seems that the relationship between OCP use and the risk of thyroid cancer is complex and can be affected by various factors such as age, the formulation of OCP pills, and genetic predisposition [38, 39].

It is important to note here that ORs may exaggerate the effect of variables, especially in cases where the inclusion of multiple confounding variables reduces the cell counts in 2 × 2 tables. This imbalance can lead to inflated OR values, which must be interpreted with caution.

### Head and neck radiation

The current study findings revealed that a history of head and neck radiation elevates the risk of thyroid cancer by over 6 times in both case‒relative control and case‒non-relative control comparisons. Exposure to ionizing radiation, especially on the human body's cervical region and thyroid gland, notably through X-rays, is commonly acknowledged as a substantial and well-established risk factor for the thyroid cancer [28–30]. The aftermath of Chernobyl incident proved that that individuals, particularly children, exposed to radiation in the head and neck region frequently develop PTC [42]. Meanwhile, a study exploring the relationship between dental x-rays and the risk of thyroid cancer highlighted a significant association between self-reported dental X-ray exposure, particularly with repeated exposures, and the risk of thyroid cancer. In this study, it was shown that there is a dose–response pattern for this association [43]. According to the literature, ionizing radiation interacts with DNA, leading to DNA strand breaks at vulnerable sites and somatic mutations, and ultimately triggering carcinogenesis, positioning it as the most consistent risk factor for TC [44, 45]. In the present study, due to the closeness of the OR values in case‒relative control relationship compared to case‒non-relative control relationship, it seems that recall bias plays a minor role in these associations and exposure to radiation in the head and neck region acts as a real risk factor for thyroid cancer. In the present investigation, the proximity of the odds ratio values in the case-relative control comparison to the case-non-relative control comparison suggests that recall bias minimally influences these relationships, emphasizing that radiation exposure in the head and neck region indeed serves as a genuine risk factor for thyroid cancer.

### Thyroid nodule

The history of thyroid nodule remained in the regression model pertaining to the relative control group with an odds ratio of 8.57 (the highest OR), signifying that a prior history of benign thyroid nodule amplifies the odds of thyroid cancer by 8.57 times. However, in the non-relative control group, this factor was not remained in the model and seemed not to contribute to creation of the best model. One plausible interpretation of this occurrence is that individuals with thyroid nodules receive frequent follow-ups at short intervals. For benign thyroid nodules, it is crucial to maintain consistent follow-up with a healthcare provider. Research findings indicate that the majority of benign thyroid nodules demonstrate a favorable prognosis, accompanied by a minimal likelihood of progressing into thyroid cancer throughout time. Nonetheless, healthcare professionals continue to assess the dimensions and attributes of the nodule through regular ultrasound evaluations and/or Fine Needle Aspiration (FNA) procedures conducted under ultrasound guidance to detect any alterations warranting further medical intervention. For this particular reason, the likelihood of detecting thyroid cancer escalates within this category of people. In addition to above explanation, empirical evidence suggests that certain nodules initially classified as benign may progress to a malignant state over time. In light of the aforementioned rationales, it appears that previous occurrence of a benign thyroid nodule may not pose a significant risk factor unless the presence of dysplasia or malignancy indications.

### Job

The results of current investigation illustrated that housekeeping emerged as the second most important risk factor with a notable odds ratio in case-non relative control comparison, so that being a housekeeper increased the chance of thyroid cancer by 40 times than being an employee. While being self-employed versus being an employee did not exhibit a significant impact on the chance of thyroid cancer.

Existing evidence suggests that housekeepers generally exhibit inferior self-care behaviors and healthcare accessibility in comparison to employed women. For instance, a study conducted on postmenopausal females in Tehran, Iran discovered a positive correlation between self-care practices and employment status, revealing that housewives displayed considerably lower self-care scores in comparison to employed women [46]. Moreover, a separate study indicated that transitioning from being a housekeeper to paid employment can yield mental health advantages for women, implying that employment status influences self-care practices and health results [47].

Consequently, based on the findings of the present study and prior research, it appears that employment and the personal income derived from it can significantly influence the utilization of healthcare services for disease prevention and early detection among women.

### Family history of cancer

The results of the present study showed that a family history of cancer, regardless of the type (thyroid/other parts of the body) significantly raises the chance of developing thyroid cancer by approximately 12 times. The relationship between family history of cancer in first-degree relatives (parents/siblings/children) has been investigated in various studies. Various research studies have explored the association between a cancer history among first-degree relatives (e.g., parents, siblings, children) and thyroid cancer. For instance, a study featured in the National Center for Biotechnology Information (NCBI) indicated that a family history of thyroid cancer among first-degree relatives was linked to an escalated risk of PTC, with an adjusted OR of 4.1 (95% CI: 1.7–9.9). Furthermore, within this particular study, the association between a history of different cancer types among relatives and thyroid cancer was examined. The outcomes highlighted that out of over 30 cancer variations, solely a family history of liver cancer exhibited an association with an increased risk of thyroid cancer, displaying an OR of 4.8 (95% CI: 1.6–14.7) [48]. Another research published in the NCBI outlined that a family history of thyroid cancer among first-degree relatives was tied to an amplified risk of PTC Notably, the risk was more pronounced in individuals with a family history of thyroid cancer among siblings, showing an OR of 7.4 (95% CI: 1.8–30.4) [49]. These results emphasizing an elevated TC risk linked to a family cancer history in first-degree relatives may suggest a genetic element in thyroid cancer's etiology.

### History of pregnancy

The examination of pregnancy history in this study unveiled that the absence of pregnancy history heightened the chance of thyroid cancer by 4.5 times in the case‒relative control comparison, whereas no substantial association was observed in the case‒non-relative control and case-total control comparisons. Based on the evidence from pertinent research, a higher number of pregnancies and early onset of menstruation (pre-12 years) are tied to an escalated risk of thyroid cancer [50]. In addition, a delayed age at first pregnancy is associated with a decreased risk of thyroid cancer [51]. Literature indicates the presence of estrogen receptors in the thyroid gland, influencing the biology of thyroid tumors [52, 53]. Consequently, thyroid cell proliferation rates may surge during elevated estrogen levels in pregnancy [52, 54]. In addition, an older age at initial pregnancy diminishes the risk of thyroid cancer by reducing thyroid cell exposure to high levels of estrogen [50]. Hence, pregnancy could potentially serve as a risk factor for thyroid cancer. This is while in the present study, pregnancy emerged as a protective factor for the occurrence of thyroid cancer in the relative control group. Nonetheless, the rationale behind this phenomenon remains unclear, necessitating further investigation to comprehensively grasp pregnancy's impact on thyroid cancer susceptibility.

A possible limitation of current research is that the association of OCP/radiation to head and neck and PTC found in the case–control study is not causal and there are neither genetic polymorphisms (SNP) nor biomarkers linked with this association. Another limitation is that it is not clear from the presented data regarding exposure to radiation due to X-Ray or CT scan, like how many of the cases or controls were exposed to either of CT scan or plain X-Ray. The exposure to CT scan would lead to greater dosage of radiation and hence more risk. The dose response relationship is vital to establish cause-effect relationship hence it wasn’t possible to detect total dose exposure in present study.

## Conclusion and future perspective

In present investigation, the utilization of OCP as well as exposure to head and neck radiation were determined to be strong or relatively strong risk factors in both case‒relative control and case‒non-relative control comparisons. Consequently, given that both variables were acknowledged as risk factors in both comparisons, it appears that irrespective of recall bias and genetic influences, these two factors represent genuine risk factors for papillary thyroid cancer.

Therefore, it is highly advisable to utilize a thyroid protective shield whenever administering X-ray in the head and neck region. It is also recommended for individuals to undergo screening for thyroid problems prior to and while using OCPs. Given the constraints of this particular study on the correlation between OCPs dosage and PTC occurrence, as well as the frequency of required examinations during OCP usage, it is proposed that a more in-depth study be conducted to explore the association between OCP usage and thyroid cancer. Meanwhile, environmental risk factors could not be explored in this research due to certain constraints. Considering the presence of numerous industrial facilities such as tile factories, as well as various mines including those extracting different metals and uranium within Yazd province, it is recommended that a study be devised to investigate the impact of environmental risk factors in residential areas, and to assess the influence of different mines, multiple factories, and pollution in the air and water.

## Supplementary Information

Supplementary Material 1.

Supplementary Material 2.

## Data Availability

The data is not available publicly. However, upon a reasonable request, the data can be obtained from the corresponding author.
