# Supplementary material for: Investigating papillary thyroid cancer risk factors among women living at the central region of Iran: a case–control study
Source: BMC Endocr Disord. 2025 Jan 16;25:12. doi: 10.1186/s12902-025-01833-3 (PMC11737264; doi:10.1186/s12902-025-01833-3)
Supplement: Supplementary file 1 — Supplementary Material 1. [file 12902_2025_1833_MOESM1_ESM.docx]

**Data collection form**

Group: case□ relative□ non-relative□

1. General information

Age: ………………. Education: ……………

Job: ………………..

Marital status: married □ single □ widow □ divorced □

Weight: …………. Height: ………….

Blood group: A□ B □ AB□ O□

1. Questions on reproductive system

1) Age at the onset of menstruation: .....

2) Have your periods always/often been regular? Yes□ No□

3) Have you reached menopause? Yes□ No□

4) Do you take birth control pills: Yes□ No□

1. Questions related to married women

5) Have you ever been pregnant? Yes□ No□

6) Have you ever had a miscarriage? Yes□ No□

7) Have you ever had a live birth? Yes□ No□

1. Questions related to other diseases/drug use

8) Have you ever had a benign mass (nodule/adenoma) in the thyroid? Yes□ No□

9) Have you ever had goiter? Yes□ No□

10) Have you ever had hyperthyroidism? Yes□ No□

11) Have you ever had hypothyroidism? Yes□ No□

12) Have you ever had any diseases other than thyroid disease: Yes□ No□

13) Have you ever had history of head and neck radiation: Yes□ No□

14) Have you ever had a history of cancer in first degree family: Yes□ No□
