# Supplementary material for: Investigating papillary thyroid cancer risk factors among women living at the central region of Iran: a case–control study
Source: BMC Endocr Disord. 2025 Jan 16;25:12. doi: 10.1186/s12902-025-01833-3 (PMC11737264; doi:10.1186/s12902-025-01833-3)
Supplement: Supplementary file 2 — Supplementary Material 2. [file 12902_2025_1833_MOESM2_ESM.docx]

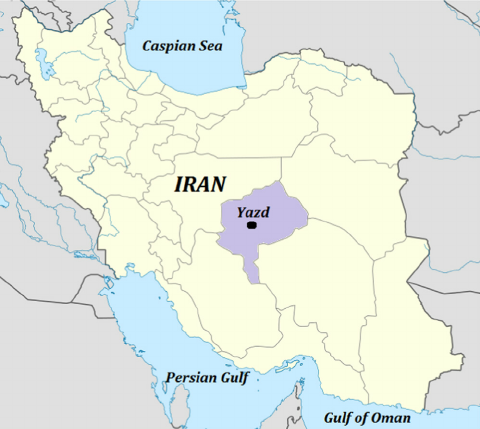


Supplementary Figure 1. Yazd province map*.

* The presented map has been obtained from this address: https://www.researchgate.net/figure/Location-of-Yazd-city-on-the-map-of-Iran-Yazd-province-is-shown-in-highlight_fig2_263088654
